# Exposure to a Vitamin D Best Practices Toolkit, Model, and E-Tools Increases Knowledge, Confidence, and the Translation of Research to Public Health and Practice †

**DOI:** 10.3390/nu15112446

**Published:** 2023-05-24

**Authors:** Beth S. Sanford, Jennifer L. Aliano, Courtney S. Omary, Sharon L. McDonnell, Samantha M. Kimball, William B. Grant

**Affiliations:** 1 School of Nursing, Rasmussen University, 4012 19th Avenue South, Fargo, ND 58103, USA; 2 GrassrootsHealth Nutrient Research Institute, Encinitas, CA 92024, USA; jen@grassrootshealth.org (J.L.A.); samantha@grassrootshealth.org (S.M.K.); 3 Sunlight, Nutrition, and Health Research Center, San Francisco, CA 94164-1603, USA; wbgrant@infionline.net

**Keywords:** vitamin D, vitamin D deficiency, toolkit, model, patient care technology, e-tools, evidence-based practice, public health, global health, continuing education, healthcare professional education

## Abstract

Preventable vitamin D deficiency (VDD) is a global health concern. The prevention, early detection, and treatment of vitamin D deficiency aligning with serum 25-hydroxyvitamin D concentration recommendations of 40–60 ng/mL (100–150 nmol/L), provided by an international panel of 48 vitamin D researchers, would result in significant health benefits and cost savings to individuals and society. However, research shows that healthcare professionals lack knowledge and confidence in best practices with respect to vitamin D. A vitamin D toolkit was developed that included a model for decision-making support, e-tools, and accompanying resources and was implemented using an online, asynchronous learning management system. This pre-test, post-test, and follow-up survey study design aimed to increase nurses’ and dietitians’ levels of knowledge and confidence regarding vitamin D, aid in their translation of evidence into spheres of practice and influence, and help them identify translation barriers. The completion of the toolkit increased the participants’ (*n* = 119) knowledge from 31% to 65% (*p* < 0.001) and their confidence from 2.0 to 3.3 (*p* < 0.001) on a scale of 1–5. Respondents reported using the model (100%) as a framework to successfully guide the translation of vitamin D knowledge into their sphere of influence or practice (94%) and identifying translation barriers. The toolkit should be included in interdisciplinary continuing education, research/quality improvement initiatives, healthcare policy, and institutions of higher learning to increase the movement of research into practice.

## 1. Introduction

An opportunity exists to educate the public, healthcare professionals (HCPs), and policymakers on the global epidemic of vitamin D deficiency (VDD), current vitamin D science and physiological mechanisms, the health benefits and cost-effectiveness of achieving and maintaining the scientifically recommended circulating 25-hydroxyvitamin D [25(OH)D] concentrations of 40–60 ng/mL (100–150 nmol/L), and proper nutrient study design [1,2,3]. Since the early 2000s, researchers have studied the relationship between VDD, patient outcomes, and overall healthcare costs [4,5,6,7,8,9,10,11]. Studies conducted by the United States Veterans Affairs Administration have demonstrated an overall 39% increase in healthcare costs and adverse patient outcomes associated with serum 25(OH)D concentrations <20 ng/mL [4]. From a public health perspective, the cost-effectiveness of the prevention, early detection, and treatment of VDD would result in significant cost-savings to individuals and society as well as reduce human suffering [12,13].

Research shows that HCPs’ knowledge and attitudes affect their behaviors in addressing VDD [14]. In light of comments in personal conversations, on social media, in mass media, opinion pieces, and vitamin D research articles and through a critical appraisal of published vitamin D study designs, guidance in implementing evidence-based best practices regarding vitamin D in practice is necessary to achieve the quadruple aim of healthcare related to preventable VDD (patient experience, reducing costs, improving patient and population health outcomes, and improving the HCP experience) [15].

Evidence-based practice is a critical thinking process that incorporates the review, analysis, and translation of the most current science, practice, and patient or population risk factors and determinants of health to guide public health and clinical decision making [16]. The scientific literature, however, is lacking in models and translational resources to aid the movement of vitamin D science into practice. The development of better clinical guidelines, electronic decision aids, and visual cues has been recommended to aid the ease of moving vitamin D research into practice [17,18,19,20]. Further education and training were also recommended to increase both students’ and practicing HCPs’ vitamin D knowledge and increase the translation of this knowledge into practice and public health [21,22,23,24,25,26,27,28,29,30]. Research shows that HCP education increases knowledge, results in feelings of enhanced self-efficacy, and increases the sustainability of evidence-based practice (EBP); however, authors have noted that translational methods are needed to sustain a long-term practice change [31]. Providing decision-making support in the form of a translational model and best practice resources may fill a gap in HCPs’ education needs, resulting in increased knowledge about vitamin D and increased opportunities for policy and practice change [27,32,33].

The aim of this paper is to introduce an evidence-based toolkit, including a translational model for best practices for addressing vitamin D deficiency in public health and practice, highlighting existing evidence-based patient care technologies, and providing key resources to enhance the knowledge and confidence of health policy makers and HCPs when addressing VDD in patients and populations from primary, secondary, or tertiary prevention perspectives.

### 1.1. Vitamin D Overview

Vitamin D is critical to cellular health, and nearly every cell in the human body has a vitamin D receptor (VDR), which is activated by calcitriol [34,35]. Vitamin D has several forms that are critical to the biochemical functions of cellular health and organ systems. Vitamin D compounds function as a vitamin–nutrient, cell-signaling molecule, prehormone, or hormone, depending on the molecular structure, function, and place in the life cycle of vitamin D [36,37,38].

Vitamin D plays a significant role in most physiologic and pathologic processes in the body, including DNA repair mechanisms, the prevention of cancer cell proliferation, lowering viral replication rates, antimicrobial defenses, lowering pro-inflammatory cytokine concentrations, the balance of the microbiota, innate and adaptive immune system homeostasis, and gene expression [35,39,40,41]. A 2019 randomized controlled double-blind clinical trial (RCT) demonstrated a dose-dependent relationship between vitamin D3 supplementation and gene expression, showing the up- or down-regulation of 1289 identified genes with the daily supplementation of 10,000 IU/day compared to 320 genes with the supplementation of 4000 IU/day and 162 genes with the supplementation of 600 IU/day [42].

### 1.2. Totality of Evidence

VDD is 100% preventable and is a modifiable risk factor for many health concerns. Nutritional rickets is still endemic in many countries; however, experts agree that rickets is “the tip of the iceberg of VDD disorders” [43]. Scientists have been studying the nonskeletal effects of vitamin D on conditions such as cancer, insulin secretion, and brain and cardiovascular health since the early 1980s [44,45,46]. Since the year 2000, however, vitamin D science has advanced significantly beyond its skeletal effects. There are four recent papers that provide a thorough overview of current vitamin D science, including a rationale for the poor results found by some RCTs [1,2,47,48].

As discussed in detail in Grant et al., the Hill criteria for causality have been analyzed in relation to VDD, and nearly all have been satisfied for many healthcare concerns, including hypertension, cancer, cardiovascular disease (CVD), COVID-19, dementia, type 2 diabetes, pancreatic cancer, type 1 diabetes, multiple sclerosis, oral health, and periodontal disease when using observational, population, and in some instances, Mendelian randomization studies [2,48,49,50]. Mendelian randomization studies have also shed light on the roles of genetics and vitamin D metabolism, showing the need for personalized approaches for the prevention and treatment of vitamin D deficiency in addition to public health measures [2].

Different cellular processes and systems within the body have differing needs for Vitamin D due to the unique physiological mechanisms and roles vitamin D plays in each system. Research has demonstrated how different health conditions may also have different 25(OH)D minimum concentrations for optimal functioning, i.e., decreased all-cause mortality, decreased stroke, decreased myocardial infarction, reductions in blood pressure and the prevalence of hypertension, decreased incidence of type 2 diabetes, improved prenatal and neonatal outcomes (including preterm birth, pre-eclampsia, and gestational diabetes), improved thyroid function, and cancer reduction benefits (see Table 1) [2,21,51,52,53,54,55,56,57,58,59,60,61,62]. These studies suggest public health agencies, institutions, and individual HCPs should leverage optimal 25(OH)D concentrations of 40–60 ng/mL (100–150 nmol/L) to achieve body systems’ minimum optimal 25(OH)D concentrations and offset individual and population risk factors for disease, such as medication use, pre-existing health conditions, lifestyle, family history, genetics, and determinants of health. Researchers have hypothesized that health disparities could be eliminated for those of African ancestry if optimal 25(OH)D concentrations were achieved and maintained [63]. Areas of particular global and public health interest include the cost-effectiveness of addressing vitamin D deficiency/insufficiency in prenatal outcomes, including increased maternal morbidity and mortality [64,65,66], and mental health, including the risk of opioid addiction [67], the risks of depression and suicide [68,69,70,71,72,73,74,75,76], and childhood behavioral problems [77], as well as COVID-19 [78] and HIV transmission [79].

### 1.3. Healthcare Professionals’ Knowledge of Best Practices

In a study on vitamin D knowledge in HCPs, only 1.7% felt that they were trained properly in how to properly diagnose and manage VDD [20]. In addition, upon evaluation, less than half of the HCPs were reported to have good practice at addressing VDD [22]. In another study on HCPs, the recommendation and dosing of supplements was inconsistent, and minimal patient education was provided with respect to lifestyle practices, sun exposure, nutrition, and diet around 50% of the time [24]. In a study on general practitioners, 97% believed clearer clinical guidelines regarding VDD would be beneficial, while roughly 35% believed it was more important to stay out of the sun than to get enough vitamin D [17]. Another study found that family practice physicians erroneously believed that sunlight alone was adequate to prevent VDD in their geographic location [18]. A study among pharmacists and doctors found that 41.9% and 39.3% had poor knowledge of vitamin D, 47.5%, 49.5% had poor attitudes, and 44.1% and 39.3% had poor knowledge of nutrition, respectively [82]. In a study of doctors caring for pregnant women, 35% did not assess women for VDD, and 32% only assessed high-risk women [83]. In a study on pediatricians’ knowledge of vitamin D best practices, participants were least knowledgeable with respect to when to initiate vitamin D therapy and which dosages should be used to treat low 25(OH)D concentrations [30]. In the same study, the authors noted a discrepancy between guidelines and participants’ knowledge, proposing that pediatricians may be fearful of intoxication and unclear between general supplementation guidelines and the therapeutic doses necessary for repletion [30]. In the United States, a 2009–2016 data analysis showed that less than 40% of breastfeeding and non-breastfeeding infants between the ages of 0 and 11 months met the American Academy of Pediatrics guidelines for vitamin D supplementation (≥400 IU) regardless of demographic subgroups [84].

### 1.4. Professional Organizations as a Platform for Disseminating Evidence-Based Practice

Professional organizations are a reliable source of health information for HCPs and are a key venue for introducing practice changes [85]. Professional organizations can aid in reducing health disparities by creating focused health initiatives to promote EBP through the dissemination of scientific discoveries and translational support materials on multiple levels of social-ecological models. State or regional professional organizations can guide the translation of EBP to the local cultural context and determinants of health. EBP can be disseminated through many avenues, such as encouraging involvement in professional organizations, networking among members, and hosting continuing education seminars/webinars, poster presentations, and conferences [85,86].

## 2. Materials

### 2.1. Toolkit Development

The toolkit was designed to be a self-paced, interactive learning experience accommodating different types of adult learners. The principles of Bandura’s social cognitive theory were used to create anticipation for an increase in self-efficacy after completing the e-course portion of the toolkit [87]. The e-course contains an introduction, learning objectives, and modules on vitamin D science, including vitamin D myths, critical appraisal of nutrient research, the cost-effectiveness of vitamin D, the science behind optimal 25(OH)D concentrations, supplementation dosing, toxicity and testing, and the consequences of VDD [88].

In addition, the toolkit guides the translation of research into practice by integrating a “best practices” model (described below), the Socioecological Model, as adapted by Golden [89], and levels of prevention as guiding frameworks. An abundance of downloadable peer-reviewed journal articles and translation resources such as e-tools accompanied the e-course as part of the toolkit to encourage the translation of research into practice. Subject matter experts established the face validity of participant assessments, the model, and all educational and translational content.

### 2.2. Model Development

**Figure 1:**
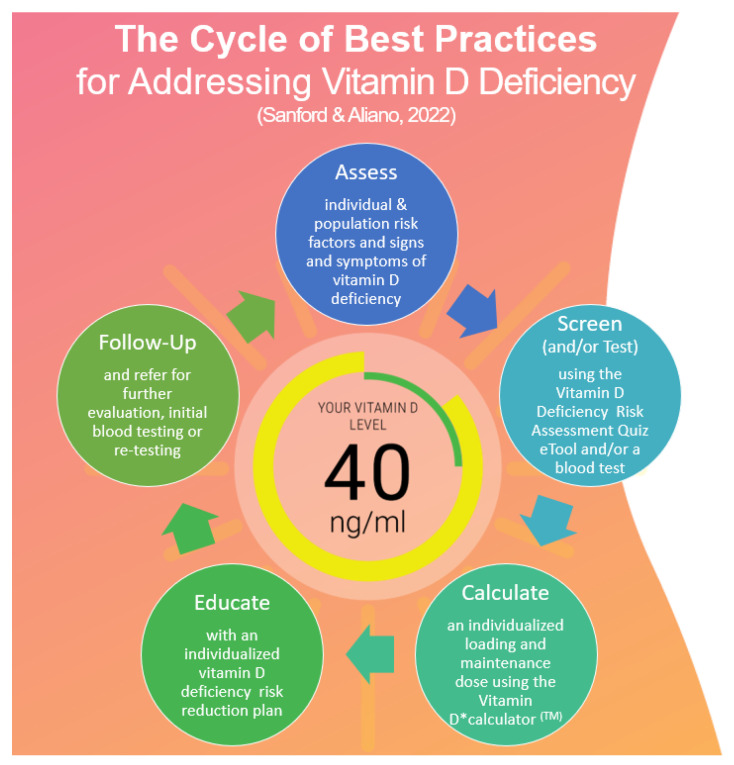
The Cycle of Best Practices for Addressing Vitamin D Deficiency Model [92] The evidence-based e-tools: Vitamin D Deficiency Risk Assessment Quiz (beta) and Vitamin D*calculator^TM^, and IRB-approved Know “D” Number: Patient and Provider Guide to Understanding Vitamin D, Testing and Results can be found at https://grassrootshealth.net/project/achieve-manage-optimal-vitamin-d-levels/ (accessed on 18 April 2023).

The Cycle of Best Practices for Addressing Vitamin D Deficiency (the model), was developed as a new paradigm for patient-centered care and standards of best practices for vitamin D deficiency based on vitamin D science, GrassrootsHealth’s D*Action Project vitamin D protocol, and the Screening, Brief Intervention, and Referral to Treatment (SBIRT) model (see Figure 1 below) [90,91,92]. The GrassrootsHealth D*Action Project protocol (also used in research on pregnancy outcomes at the Medical University of South Carolina) includes initial testing, vitamin D3 supplementation, patient education, retesting, and the monitoring of patient outcomes, targeting 25(OH)D concentrations of >40 ng/mL [93]. The SBIRT model is a public health approach to early intervention and treatment that encourages a brief screening, intervention, and referral to follow-up treatment, a model that is feasible and sustainable for HCPs [91].

#### 2.2.1. Model Components

The model is based on a five-step process designed to educate the patient and the provider alike in the steps necessary to achieve and maintain circulating target 25(OH)D concentrations safely and efficiently. The steps are (1) assess, (2) screen (or test), (3) calculate, (4) educate, and (5) refer.

##### Assess

Preliminary vitamin D research findings demonstrate that the individual response to the same vitamin D supplement dose or sun exposure practices can vary up to six times [94]. Therefore, individualized patient care is recommended when assessing individual and population risk factors as well as signs and symptoms of deficiency [95]. Individual risk factors include but are not limited to: medication use, sun exposure practices, skin color, genetics, absorption factors, vegan/vegetarian diet, and co-nutrient status, such as magnesium and essential fatty acids [39,63,96]. A free infographic, Everyone Responds Differently to Vitamin D, can aid healthcare professionals in assessing individual patient risk and can be used as a tool in primary prevention education initiatives [97].

##### Screen (or Test)

Screening for vitamin D deficiency risk level can now be completed using a non-invasive patient care technology, now in beta mode, called the Vitamin D Deficiency Risk Assessment Quiz (beta) [98]. Preliminary results from quiz testing show that the quiz can determine an individual’s risk of having a 25(OH)D concentration below the recommended minimum of 40 ng/mL with a score of a low, medium, or high risk. These results may be useful to healthcare professionals in determining the need for a 25(OH)D blood test referral, depending on clinical indications [3]. A 25(OH)D blood test screening is strongly recommended for at-risk populations, such as pregnant women, or those with limited sun exposure for any reason.

##### Calculate

Calculating an individualized vitamin D supplement maintenance dose and loading dose, if needed, to maintain an optimal 25(OH)D concentration can be achieved with confidence using a patient care technology: the evidence-based Vitamin D*calculator™, which is based on an evidence-based formula [99]. An individualized patient vitamin D supplementation dose can be calculated using the current 25(OH)D concentration, target 25(OH)D concentration, and the patient’s current weight. The Vitamin D*calculator will soon also incorporate age into the dosing formula.

##### Educate

Upon completion of the Vitamin D Deficiency Risk Assessment Quiz, patient risk factors are highlighted for individualized patient education points. The results can easily be emailed to the patient and healthcare professional for reference.

An individualized vitamin D deficiency risk reduction plan should include:

- Addressing individual patient risk factors.
- Incorporating a safe sun or UVB exposure routine based on skin type, lifestyle, seasonality, and environmental determinants of health that affect the UV index, such as latitude, pollution, and inclement weather.
- Maintaining a healthy diet to maximize vitamin D absorption and the supplementation of necessary co-nutrients such as magnesium, K2, and essential fatty acids.
- Providing education on the individualized vitamin D3 supplementation dosing routine, as outlined by the vitamin D*calculator™ recommendations.
- Providing high-quality patient education materials written by vitamin D researchers, such as the GrassrootsHealth Nutrient Research Institute’s IRB-approved “Know ’D‘ Number: Patient and Provider Guide to Understanding Vitamin D, Testing & Results” [100].

##### Follow Up

Healthcare professionals should consider a referral for follow-up 25(OH)D testing in 3–6 months to determine if the recommended dose was successful at achieving the target concentration of 40–60 ng/mL. Retests should then occur retest annually or as needed to maintain optimal blood concentrations.

## 3. Methods

### 3.1. Setting and Participants

The project leveraged a relationship with the North Dakota Nurses Association and the North Dakota Academy of Nutrition and Dietetics (NDNA and NDAND) for its implementation, focusing on nurses and dietitians as target participants. The NDNA and NDAND maintain an active website and social media presence, including continuing education opportunities. Both organizations publish periodic email and paper newsletters and post social media updates. The link for the course was made available through publications brought forth by the NDNA and NDAND, including newsletters, emails, and social media. Reminders and advertising supported recruitment and completion of the project to access eligible participants.

### 3.2. Toolkit Design and Use Process

Following knowledge synthesis, an online, asynchronous toolkit was developed using an internet-based learning management platform. The toolkit content included a vitamin D education e-course, The Cycle of Best Practices for Addressing Vitamin D Deficiency model (model) [92], evidence-based e-tools to aid HCPs in translating research to practice, and downloadable research and translation resources for patient and provider education, such as “KNOW ‘D’ NUMBER Patient and Provider Guide to Understanding Vitamin D, Testing, and Results” [100].

After participants were introduced to the toolkit and learning objectives, they confirmed their consent to participate in the project and moved directly onto the pretest. The pretest included demographic questions and ten questions capturing baseline vitamin D knowledge. Participants then moved through the educational modules and were assessed with the same ten questions during the posttest, followed by a toolkit satisfaction survey. The learning management system captured the participants’ consent and responses [101]. Post test, the participants rated the delivery, time, and ease of the training on a ten-point Likert-style scale.

Two weeks post education, a follow-up survey was sent to those participants who completed the entire knowledge assessment. Follow-up questions assessed participants’ confidence, the use of the model and best practice resources in their sphere of practice and influence, as well as perceived barriers to moving research into practice.

The use of the model was tested during the follow-up survey two weeks after the completion of the vitamin D toolkit. The participants’ use of the model was evaluated using the following question: “Since finishing the course, I have utilized the following components of (the model) within my sphere of influence (select all that apply)”: Assess, Screen (or Test), Calculate, Educate, Refer.

### 3.3. Statistics

The following statistical tests were used in this project: demographic frequencies; knowledge scores: pre vs. post—paired, two-tailed *t*-test; and confidence levels (Likert Scale 1–5): pre vs. post—paired, two-tailed *t*-test.

## 4. Results

### 4.1. Participant Demographics

The pretest, educational content, and posttest were completed by 119 participants. Eighty-seven participants (73%) completed the two-week post-intervention follow-up survey. On average, participants took 1.5 h to complete the toolkit. Of the participants completing the toolkit, 86% were nurses (*n* = 112) and 13% (*n* = 16) were dietitians (see Table 2). One participant chose not to disclose their healthcare discipline. The educational degree of the participants included bachelor’s degrees, 45% (*n* = 54); master’s degrees, 23% (*n* = 27); licensed practical nurses, 14% (*n* = 23); associate degrees, 12% (*n* = 14); and doctoral degrees, 5% (*n* = 6) (see Table 3). The largest group of participants were registered nurses with bachelor’s degrees (36%). When asked about their retirement and employment status, most participants indicated they were not retired (96%) and were currently employed as healthcare professionals (91%). The demographics of the follow-up survey (*n* = 86) were as follows: 83% nurses, 16% dietitians, and 1% other (see Table 4).

### 4.2. Knowledge Assessment Results

Through the use of a paired *t*-test, the mean pre-post knowledge scores (*n* = 119) showed a statistically significant increase from 31% to 65% (*p* < 0.0001) (see Table 5). The sample size was too small to carry out subgroup analyses between nurses and dietitians. 

### 4.3. Toolkit Feedback

The participants provided feedback about the course: (1) “The course contained a ton of research-based information and resources. I was amazed at the data and how nicely it was compiled in this format. Well done!! Although I haven’t had much time yet to pass along the information since taking the course, I definitely plan to!” (2) “Thought the course was well done, format easy to follow, provided actionable steps.” (3) “I was impressed with the quantity and quality of resources. Thank you for sharing! I plan to use some of the materials in my teaching practice”.

### 4.4. Follow-Up Survey Results

**Figure 2:**
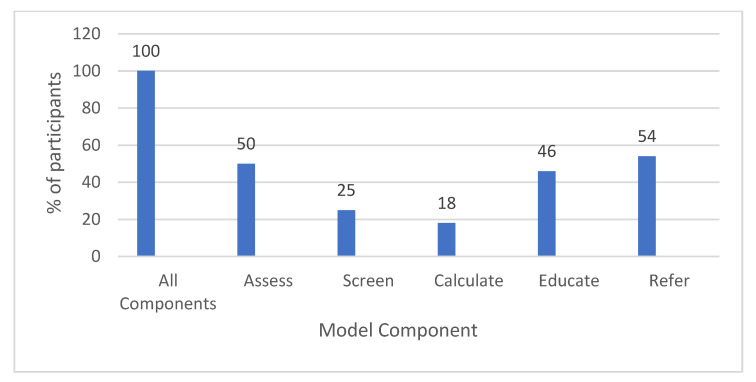
Percent of participants who used a component of the Cycle of Best Practices for Addressing Vitamin D Deficiency within their sphere of influence or practice (*n* = 72).

**Figure 3:**
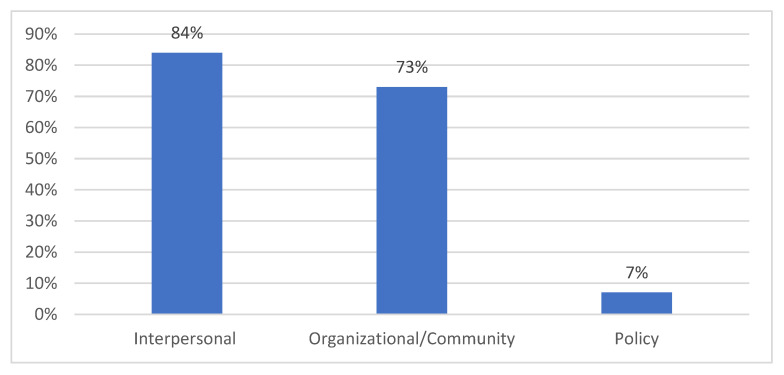
Translation of research into practice or sphere of influence, shared by SEM level.

The follow-up survey demonstrated five outcomes:

(1) Increased confidence in translating research to practice: participants (*n* = 86) reported increased confidence in translating research to practice. A paired *t*-test showed that the participants’ confidence scores increased significantly, from 2.0 to 3.3 on a scale of 1–5 (*p* < 0.0001) (see Table 6).
(2) Follow-up survey results found that 100% of the follow-up participants (*n* = 72) reported translating research into practice within their sphere of influence or practice using at least one component of the model. The most commonly used model components were as follows: refer (54%), assess (50%), educate (46%), screen (25%), and calculate (18%), respectively (see Figure 2).
(3) The translation of research into practice or sphere of influence: of the participants, 94% (*n* = 85) shared knowledge within their practice or sphere of influence, with the most common socio-ecological model (SEM) levels being:
  - Interpersonal (friends, family, and patients): 84%
  - Organizational/community (coworkers and community members): 73%
  - Policy (professional organization members, legislators, or health department staff): 7% (see Figure 3)
(4) The most reported resource used to translate research into practice was the “Know “D” NUMBER: Patient and Provider Guide to Understanding Vitamin D, Testing and Results” [100].
(5) The participants reported that the most perceived barrier to translating vitamin D knowledge into practice were financial barriers, including the cost of testing and the lack of insurance coverage. Other identified barriers included resistance from interdisciplinary team members and individuals or the patients’ lack of interest in vitamin D information.

## 5. Discussion

This pilot project successfully synthesized existing vitamin D science and research into an easily accessible toolkit and public health translational model with key resources that increased HCP knowledge and confidence in translating best practices and verified barriers to moving research into practice. The toolkit makes years of vitamin D research readily available and digestible to HCPs who are looking to understand evidence-based best practices with respect to vitamin D and their role in the health of their patients and communities. The self-paced toolkit alleviates the perceived barrier that the translation of scientific literature and evidence is time-consuming [102].

The Cycle of Best Practices for Addressing Vitamin D Deficiency model was successful in guiding nurses’ and dietitians’ translation of vitamin D knowledge, gained from the completion of the vitamin D toolkit, into their spheres of influence or practice. The model provides a framework built around the understanding that in order to address VDD most accurately, it must be addressed from an individualized, patient-centered care perspective, taking into account individual risk factors, determinants of health, individualized dosing, existing 25(OH)D concentrations, and the need for follow-up 25(OH)D testing to assess the effectiveness of the vitamin D dosing regimen at achieving and maintaining optimal serum 25(OH)D concentrations.

Key findings support previous research that HCPs lack knowledge and confidence in vitamin D science and best practices. However, exposure to continuing education, a best practice model, and evidence-based resources increase HCPs’ knowledge and confidence in translating research into their practice and spheres of influence. The results demonstrate that global and public health initiatives should implement an evidence-based vitamin D toolkit, translational model, and easily accessible resources as the standard of care and policy. The toolkit, including the e-course, model, e-tools, and accompanying resources, can be easily implemented in a variety of public health and patient care settings to guide public health and policy development, research and quality improvement initiatives, clinical decision making, and patient education. Additional healthcare professional education resources, including the Vitamin D Resources for Healthcare Professionals handout and Vitamin D and Nutrient Research Links can be found in the supplementary materials for this article.

### 5.1. Utilizing Evidence-Based Patient Care Technologies

Leveraging patient care technologies, such as the following e-tools, to aid in the ease of translation may optimize workflow and increase confidence when screening patients for VDD or calculating a patient’s individualized vitamin D loading or maintenance dose, thus improving the clinician experience.

#### 5.1.1. Vitamin D Deficiency Risk Assessment Quiz

The Vitamin D Deficiency Risk Assessment Quiz (beta) is an e-tool that was developed and validated in 2022 by a team of vitamin D researchers [103]. The quiz was designed to increase awareness and knowledge about low vitamin D for patients and HCPs and conveniently screen for necessary referrals for blood testing in various patient care and community health settings when blood testing is unavailable or not affordable. The quiz results show the patient’s risk for a 25(OH)D concentration below the recommended minimum of 40 ng/mL (low, medium, or high risk) and list patient-specific risk factors based on the answers provided for use in patient education and knowledge reinforcement for the HCP. All information generated by the quiz can be automatically emailed to the patient for their records or later follow-up.

#### 5.1.2. Vitamin D*Calculator™

The Vitamin D*Calculator™ was developed and validated in 2015 and updated in 2020 by a team of vitamin D researchers using GrassrootsHealth data [104]. The calculator estimates the amount of vitamin D intake required to achieve and maintain a desired serum 25(OH)D concentration quickly and safely in the form of an optional loading dose and a daily maintenance dose. It is easy and convenient, and the maintenance dose is capped at 10,000 IU per day to match the no observed adverse effect level (NOAEL). This maintenance dose is the individualized daily dose recommended for achieving the patient- or HCP-determined target 25(OH)D concentration according to the current 25(OH)D concentration and weight based on the average vitamin D dose response of the GrassrootsHealth cohort. The calculator includes a practical loading dose regimen that would enable the rapid correction of VDD based on the following formula published by van Groningen et al. (2010): dose (IU) = 40 × (target − starting 25(OH)D concentration in nmol/L) × body weight in kilograms [105]. This formula can be converted to Dose (IU) = 40 × (target − starting 25(OH)D concentration in ng/mL) × body weight in pounds. The calculator has starting points for patients with or without a known 25(OH)D concentration and will soon include age as an additional factor. The target serum concentration can be adjusted based on individual patient needs.

## 6. Safe Sun Exposure Practices

Public health guidelines for safe sun exposure should be based upon two factors: local or regional recommendations based on determinants of health and individual factors [106]. Determinants of health such as latitude, altitude, inclement weather (hot or cold), and weather patterns (sunny or cloudy) all affect the UVB exposure or the body’s ability to synthesize vitamin D from solar radiation; for example, communities above 34 degrees latitude experience a vitamin D production winter in which ultraviolet-B rays (UVB) do not reach the Earth for up to six months of the year, limiting the population’s total annual sun exposure [106,107]. Likewise, harsh climates with inclement weather, either too hot or too cold, drive the population indoors, while excess cloud cover can block up to 99% of UVB [108]. Safe sun exposure should be encouraged as long as individuals take care not to burn, acclimating from the winter to the spring and summer months, and are mindful of following the “shadow rule”, which ensures that the solar angle lies below 45 degrees [106,109].

Individual factors such as skin tone, obesity, age, sun protection (sunscreen or clothing use), and cultural and lifestyle practices also affect the adequate amount of sun exposure needed to generate vitamin D [66]. If individuals stay in the sun long enough to generate a large dose of vitamin D, they risk sunburn and sun damage; therefore, individualized patient education based on their skin tone in reference to the Fitzpatrick scale is recommended [106]. For example an individual with a darker skin tone (Fitzpatrick IV) may require up to six times as much time in the sun to generate the same amount of vitamin D as an individual with the lightest of skin tones (Fitzpatrick I) [106]. Therefore, darker-skinned populations living outside of their ancestral lands are at an increased risk for VDD and insufficiency. Researchers have hypothesized that health disparities could be eliminated for individuals of African ancestry if optimal serum 25(OH)D concentrations were achieved and maintained [63]. Therefore, educating the public as to safe sun exposure practices is imperative to generate vitamin D naturally when possible.

## 7. Limitations

A limitation of testing this model was that the number of participants who completed this particular follow-up survey question was small (*n* = 72). Another limitation of this pilot project was that the participants included nurses and dietitians only in the state of North Dakota and may not be representative of the interdisciplinary team as a whole or other populations of nurses and dietitians. Relying on newsletters, email advertising, and social media may have been a barrier to recruiting participants. In addition, participants may not have had the interest or time to complete the toolkit, download and use the resources, or complete the follow-up survey. Nurses and dietitians may also have anticipated challenges or barriers in implementing the knowledge. The time window between the education intervention and the follow-up survey was only two weeks; therefore, it may be challenging to project participants’ long-term knowledge retention, confidence, and use of the translational model, e-tools, and resources within their practice and sphere of influence.

## 8. Recommendations for Continuing Education, Research and Quality Improvement

Recommendations for further research include the implementation of the toolkit, with the e-course, model, e-tools, and translational resources, in a variety of interdisciplinary policymaking, educational, public health, and patient care settings to facilitate the movement of vitamin D research into practice. Additional recommendations include the inclusion of the toolkit as pre-education to guide the standardization of vitamin D research and quality improvement initiative design capitalizing on nutrient physiology per the Heaney criteria, nutrient study guidelines that capture initial serum 25(OH)D concentrations with intent to achieve and maintain optimal target 25(OH)D concentrations of 40–60 ng/mL (100–150 nmol/L), thus strengthening such initiatives as well as allowing for easier systematic analysis and review [110].

## 9. Conclusions

Addressing preventable VDD is a global health priority. Since the last vitamin D guidelines were updated, the totality of evidence available regarding vitamin D’s role in human physiology and its impact on patient outcomes and population health has increased significantly. Vitamin D researchers recommend moving a new paradigm for preventing and addressing vitamin D deficiency into practice and policy: individualized patient care based on local or regional determinants of health, population risk factors, and patient medical history and personal health concerns, with the intent to sustain optimal 25(OH)D concentrations of 40–60 ng/mL (100–150 nmol/L). Focused local, regional, or national vitamin D and safe sun exposure initiatives have the potential to significantly improve patient outcomes, population health, and healthcare professional satisfaction while decreasing healthcare costs.

This successful pilot demonstrates that the use of a vitamin D toolkit increased nurses’ and dietitians’ knowledge, confidence, and translation of vitamin D science and best practices. The strengths of the toolkit include its ease and convenience for dissemination in an online, asynchronous, self-paced format and its included translational resources: the e-course, The Cycle of Best Practices Addressing Vitamin D Deficiency translational model, and e-tools: The Vitamin D Deficiency Risk Assessment Quiz and Vitamin D*calculator™ [99,100,102]. The priority dissemination of the vitamin D toolkit should focus on policy-making workgroups, professional organizations, institutions of higher learning, and patient care settings to impact the creation of healthcare policy and public health initiatives, guide the design of vitamin D research and quality improvement projects, introduce clinical decision-making tools, and reinforce best practices to healthcare professionals at all levels of patient care. Implementing the vitamin D toolkit into all socioecological levels may successfully prevent, detect, and resolve the issues of VDD.

## Figures and Tables

**Table 1:** Optimal 25(OH)D concentration for various body systems/conditions.

Body System/Condition	Optimal 25(OH)D Concentration	Findings	Reference
All-cause mortality	≥30–36 ng/mL	HR 1.9 (95% confidence interval = 1.6, 2.2; *p* < 0.001)	Garland and Grant, 2014 [51]
Stroke	≥30 ng/mL	aHR 1.85 (95% CI, 1.17–2.93) <20 ng/mL vs. >30 ng/mL	Judd et al. [52]
Hypertension	≥40 ng/mL	Lowered BP and reduced the prevalence of hypertension among hypertensive patients.	Mirhosseini et al. [57]
Myocardial infarction	≥30 ng/mL		Acharya et al. [53]
Type 2 diabetes from prediabetes	≥40 ng/mL		Pittas et al. [80]
Cancer, all-cause	≥40 ng/mL	Women with concentrations ≥40 ng/mL had a 67% lower risk of cancer than women with concentrations <20 ng/mL (HR = 0.33, 95% CI = 0.12–0.90).	McDonnell et al. [62]
Breast Cancer	≥60 ng/mL	Women with concentrations ≥60 ng/mL had an 80% lower risk of breast cancer than women with concentrations <20 ng/mL (HR = 0.20, *p* = 0.03).	McDonnell et al. [81]
Preterm birth	≥40 ng/mL		McDonnell et al. [21]
Thyroid function	≥50 ng/mL		Mirhosseini et al. [56]
Alzheimer’s disease/dementia/brain health	≥30 ng/mL		Grant et al. [48]
COVID-19	50 ng/mL		Gibbons et al. [59] Kaufman et al. [60]
Autoimmune disease	≥30 ng/mL	Concentrations of 40–60 ng/mL may be needed for optimal risk reduction.	Sîrbe et al. [61]

**Table 2:** Toolkit Participant Characteristics by Healthcare Discipline (N = 119).

Healthcare Discipline	N	Percent
Nurses (RN/LPN)	102	86%
Dietitians (LDN, LRN)	16	13%
Did not disclose	1	1%

**Table 3:** Toolkit participant characteristics by educational degree (N = 86).

Educational Degree	N	Percent
Licensed Practical Nurse	17	14%
Associate Degree	14	12%
Bachelor’s Degree	54	45%
Master’s Degree	27	23%
Doctoral Degree	6	5%
Prefer not to state	1	1%

**Table 4:** Follow-up survey characteristics (N = 86).

Healthcare Discipline	N	Percent
Nurses (RN/LPN)	72	83%
Dietitians (LDN, LRN)	14	16%
Did not disclose	1	1%

**Table 5:** Knowledge assessment results (*n* = 119).

Knowledge Scores	Pre-Test Score	Post-Test Score
	31%	65%

*p* < 0.0001.

**Table 6:** Participants’ confidence scores.

Confidence Scores	Pre-Score	Post-Score
	2.0	3.3

*p* < 0.0001 paired *t*-test.

## Data Availability

The data are available through the Zenodo repository and can be accessed at: https://doi.org/10.5281/zenodo.7916708.

## Supplementary Materials

The following supporting information can be downloaded at: https://www.mdpi.com/article/10.3390/nu15112446/s1, Table S1: Vitamin D Resources for Healthcare Professionals, Vitamin D and Nutrient Research Links.

## References

[1] Grant W.B., Al Anouti F., Boucher B.J., Dursun E., Gezen-Ak D., Jude E.B., Karonova T., Pludowski P. (2022). A Narrative Review of the Evidence for Variations in Serum 25-Hydroxyvitamin D Concentration Thresholds for Optimal Health. Nutrients.

[2] Grant W.B., Boucher B.J., Al Anouti F., Pilz S. (2022). Comparing the Evidence from Observational Studies and Randomized Controlled Trials for Nonskeletal Health Effects of Vitamin, D. Nutrients.

[3] Pilz S., Trummer C., Theiler-Schwetz V., Grübler M.R., Verheyen N.D., Odler B., Karras S.N., Zittermann A., März W. (2022). Critical Appraisal of Large Vitamin D Randomized Controlled Trials. Nutrients.

[4] Peiris A.N., Bailey B.A., Manning T. (2008). The relationship of vitamin D deficiency to health care costs in veterans. Mil. Med..

[5] Youssef D., Bailey B., El Abbassi A., Copeland R., Adebonojo L., Manning T., Peiris A.N. (2010). Healthcare costs of *Staphylococcus aureus* and *Clostridium difficile* infections in Veterans: Role of vitamin D deficiency. Epidemiol. Infect..

[6] Youssef D., Bailey B., El-Abbassi A., Vannoy M., Manning T., Moorman J.P., Peiris A.N. (2012). Healthcare costs of methicillin resistant *Staphylococcus aureus* and *Pseudomonas aeruginosa* infections in veterans: Role of vitamin D deficiency. Eur. J. Clin. Microbiol. Infect. Dis..

[7] Fogleman S.A., Janney C., Cialdella-Kam L., Flint J.H. (2022). Vitamin D Deficiency in the Military: It’s Time to Act!. Mil. Med..

[8] Niedermaier T., Gredner T., Kuznia S., Schöttker B., Mons U., Brenner H. (2021). Vitamin D supplementation to the older adult population in Germany has the cost-saving potential of preventing almost 30 000 cancer deaths per year. Mol. Oncol..

[9] Lacey L.F., Armstrong D.J., Royle E., Magee P., Pourshahidi L.K., Ray S., Strain J.J., McSorley E. (2022). Cost-effectiveness of vitamin D3 supplementation in older adults with vitamin D deficiency in Ireland. BMJ Nutr. Prev. Health.

[10] Hannemann A., Wallaschofski H., Nauck M., Marschall P., Flessa S., Grabe H.J., Schmidt C.O., Baumeister S.E. (2018). Vitamin D and health care costs: Results from two independent population-based cohort studies. Clin. Nutr. Edinb. Scotl..

[11] Buendía J.A., Patiño D.G. (2023). Cost-utility of vitamin D supplementation to prevent acute respiratory infections in children. Cost. Eff. Resour. Alloc..

[12] Scientists’ Call to D*action for Public Health GrassrootsHealth. https://www.grassrootshealth.net/project/our-scientists/.

[13] Baggerly C.A., Cuomo R.E., French C.B., Garland C.F., Gorham E.D., Grant W.B., Heaney R.P., Holick M.F., Hollis B.W., McDonnell S.L. (2015). Sunlight and Vitamin D: Necessary for Public Health. J. Am. Coll. Nutr..

[14] Zgliczyński W.S., Rostkowska O.M., Sarecka-Hujar B. (2021). Vitamin D Knowledge, Attitudes and Practices of Polish Medical Doctors. Nutrients.

[15] Bodenheimer T., Sinsky C. (2014). From triple to quadruple aim: Care of the patient requires care of the provider. Ann. Fam. Med..

[16] Dang D., Dearholt S., Bissett K., Ascenzi J., Whalen M. (2021). Johns Hopkins Evidence-Based Practice for Nurses and Healthcare Professionals: Model and Guidelines.

[17] Reeder A.I., Jopson J.A., Gray A.R. (2012). “Prescribing sunshine”: A national, cross-sectional survey of 1,089 New Zealand general practitioners regarding their sun exposure and vitamin D perceptions, and advice provided to patients. BMC Fam. Pract..

[18] Sherman E.M., Svec R.V. (2009). Barriers to vitamin D supplementation among military physicians. Mil. Med..

[19] Bonevski B., Girgis A., Magin P., Horton G., Brozek I., Armstrong B. (2012). Prescribing sunshine: A cross-sectional survey of 500 Australian general practitioners’ practices and attitudes about vitamin D. Int. J. Cancer.

[20] Rockwell M., Kraak V., Hulver M., Epling J. (2018). Clinical Management of Low Vitamin D: A Scoping Review of Physicians’ Practices. Nutrients.

[21] McDonnell S.L., Baggerly K.A., Baggerly C.A., Aliano J.L., French C.B., Baggerly L.L., Ebeling M.D., Rittenberg C.S., Goodier C.G., Niño J.F.M. (2017). Maternal 25(OH)D concentrations ≥40 ng/mL associated with 60% lower preterm birth risk among general obstetrical patients at an urban medical center. PLoS ONE.

[22] Mekonnen W., Feleke Y., Desalegn Y., Tarekegne G., Lambisso B., Haidar J., Zewede T. (2020). Knowledge, attitude and practice of health care workers on measuring adult vitamin D level, diagnosis of deficiency, and management of consequent health conditions in three ecologies of Ethiopia: A cross-sectional study. BMC Nutr..

[23] Al-Amri F., Gad A., Al-Habib D., Ibrahim A.K. (2017). Knowledge, Attitude and Practice Regarding Vitamin D among Primary Health Care Physicians in Riyadh City, Saudi Arabia, 2015. World J. Food Sci. Technol..

[24] Fallon E.L., Lanham-New S.A., Williams P., Ray S. (2020). An investigation of the vitamin D Knowledge, Attitudes and Practice of UK practising doctors and nurses: The D-KAP study. Proc. Nutr. Soc..

[25] Lhamo Y., Chugh P.K., Gautam S.R., Tripathi C.D. (2017). Epidemic of Vitamin D Deficiency and Its Management: Awareness among Indian Medical Undergraduates. J. Environ. Public Health.

[26] Sharma R., Mahajan R., Malhotra P. (2022). Importance of Vitamin D, Awareness and Prevention of Its Deficiency among Female Medical Students. J. Cardiovasc. Dis..

[27] Walker P., Kifley A., Kurrle S., Cameron I.D. (2019). Process outcomes of a multifaceted, interdisciplinary knowledge translation intervention in aged care: Results from the vitamin D implementation (ViDAus) study. BMC Geriatr..

[28] Nowreen N., Hameed R. (2019). Awareness regarding the importance of vitamin D and prevention of its deficiency among female undergraduate medical students. Int. J. Basic Clin. Pharmacol..

[29] Manandhar P., Manandhar N., Joshi S.K. (2021). Knowledge of Vitamin D among First-year Medical Undergraduate Students of a Medical College: A Descriptive Cross-sectional Study. J. Nepal. Med. Assoc..

[30] Petrović D., Runjić E., Buljan I., Jeličić Kadić A., Markić J. (2022). Knowledge and Practice of Pediatricians Regarding Hypovitaminosis D—A Survey across 33 European Countries. Children.

[31] van Houwelingen C.T., Ettema R.G., Bleijenberg N., van Os-Medendorp H., Kort H.S., Cate O.T. (2021). Educational intervention to increase nurses’ knowledge, self-efficacy and usage of telehealth: A multi-setting pretest-posttest study. Nurse Educ. Pract..

[32] Uko C., Utley R. (2020). Implementing Evidence-Based Vitamin D Protocol in the Dialysis Clinic: An Educational Approach. Nephrol. Nurs. J..

[33] Sanford B.S., Aliano J.L., Omary C.S., McDonnell S.L., Kimball S.M., Grant W.B. (2022). Development of a Public Health Model for Translation of Best Practices in Addressing Vitamin D Deficiency.

[34] Bikle D.D., Feingold K.R., Grunfeld C., Anawalt B., Boyce A., Chrousos G. (2000). Vitamin D: Production, Metabolism and Mechanisms of Action. Endotext.

[35] Muñoz A., Grant W.B. (2022). Vitamin D and Cancer: An Historical Overview of the Epidemiology and Mechanisms. Nutrients.

[36] Heaney R.P., Armas L.A.G. (2015). Quantifying the vitamin D economy. Nutr. Rev..

[37] Holick M.F. (2007). Vitamin D Deficiency. N. Engl. J. Med..

[38] Vieth R. (2020). Vitamin D supplementation: Cholecalciferol, calcifediol, and calcitriol. Eur. J. Clin. Nutr..

[39] Grant W.B., Al Anouti F., Moukayed M. (2020). Targeted 25-hydroxyvitamin D concentration measurements and vitamin D3 supplementation can have important patient and public health benefits. Eur. J. Clin. Nutr..

[40] Sassi F., Tamone C., D’Amelio P. (2018). Vitamin D: Nutrient, Hormone, and Immunomodulator. Nutrients.

[41] Hamza F., Daher S., Fakhoury H., Grant W., Kvietys P., Alkattan K. (2023). Immunomodulatory Properties of Vitamin D in the Intestinal and Respiratory Systems. Nutrients.

[42] Shirvani A., Kalajian T.A., Song A., Holick M.F. (2019). Disassociation of Vitamin D’s Calcemic Activity and Non-calcemic Genomic Activity and Individual Responsiveness: A Randomized Controlled Double-Blind Clinical Trial. Sci. Rep..

[43] Bouillon R., Antonio L. (2020). Nutritional rickets: Historic overview and plan for worldwide eradication. J. Steroid Biochem. Mol. Biol..

[44] Garland C.F., Garland F.C. (1980). Do sunlight and vitamin D reduce the likelihood of colon cancer?. Int. J. Epidemiol..

[45] Kadowaki S., Norman A.W. (1984). Dietary vitamin D is essential for normal insulin secretion from the perfused rat pancreas. J. Clin. Invest..

[46] Scragg R. (1981). Seasonality of cardiovascular disease mortality and the possible protective effect of ultra-violet radiation. Int. J. Epidemiol..

[47] Ghanaati S., Choukroun J., Volz U., Hueber R., Mourão C.F.D.A.B., Sader R., Kawase-Koga Y., Mazhari R., Amrein K., Meybohm P. (2020). One hundred years after Vitamin D discovery: Is there clinical evidence for supplementation doses?. Int. J. Growth Factors Stem Cells Dent..

[48] Grant W.B., Al Anouti F., Boucher B.J., Fakhoury H.M.A., Moukayed M., Pilz S., Al-Daghri N.M. (2023). Evidence That Increasing Serum 25(OH)D Concentrations to 30 ng/mL in the Kingdom of Saudi Arabia and the United Arab Emirates Could Greatly Improve Health Outcomes. Biomedicines.

[49] Hyppönen E., Vimaleswaran K.S., Zhou A. (2022). Genetic Determinants of 25-Hydroxyvitamin D Concentrations and Their Relevance to Public Health. Nutrients.

[50] Zhou A., Selvanayagam J.B., Hyppönen E. (2022). Non-linear Mendelian randomization analyses support a role for vitamin D deficiency in cardiovascular disease risk. Eur. Heart J..

[51] Garland C.F., Kim J.J., Mohr S.B., Gorham E.D., Grant W.B., Giovannucci E.L., Baggerly L., Hofflich H., Ramsdell J.W., Zeng K. (2014). Meta-analysis of all-cause mortality according to serum 25-hydroxyvitamin D. Am. J. Public Health.

[52] Judd S.E., Morgan C.J., Panwar B., Howard V.J., Wadley V.G., Jenny N.S., Kissela B.M., Gutiérrez O.M. (2016). Vitamin D deficiency and incident stroke risk in community-living black and white adults. Int. J. Stroke Off. J. Int. Stroke Soc..

[53] Acharya P., Dalia T., Ranka S., Sethi P., A Oni O., Safarova M.S., Parashara D., Gupta K., Barua R.S. (2021). The Effects of Vitamin D Supplementation and 25-Hydroxyvitamin D Levels on the Risk of Myocardial Infarction and Mortality. J. Endocr. Soc..

[54] McDonnell S., Baggerly L., French C., Heaney R., Gorham E., Holick M., Scragg R., Garland C. (2016). Incidence rate of type 2 diabetes is >50% lower in GrassrootsHealth cohort with median serum 25-hydroxyvitamin D of 41 ng/ml than in NHANES cohort with median of 22 ng/ml. J. Steroid Biochem. Mol. Biol..

[55] Mirhosseini N., Vatanparast H., Mazidi M., Kimball S.M. (2017). The Effect of Improved Serum 25-Hydroxyvitamin D Status on Glycemic Control in Diabetic Patients: A Meta-Analysis. J. Clin. Endocrinol. Metab..

[56] Mirhosseini N., Brunel L., Muscogiuri G., Kimball S. (2017). Physiological serum 25-hydroxyvitamin D concentrations are associated with improved thyroid function-observations from a community-based program. Endocrine.

[57] Mirhosseini N., Vatanparast H., Kimball S.M. (2017). The Association between Serum 25(OH)D Status and Blood Pressure in Participants of a Community-Based Program Taking Vitamin D Supplements. Nutrients.

[58] Pittas A.G., Kawahara T., Jorde R., Dawson-Hughes B., Vickery E.M., Angellotti E., Nelson J., Trikalinos T.A., Balk E.M. (2023). Vitamin D and Risk for Type 2 Diabetes in People With Prediabetes. Ann. Intern. Med..

[59] Gibbons J.B., Norton E.C., McCullough J.S., Meltzer D.O., Lavigne J., Fiedler V.C., Gibbons R.D. (2022). Association between vitamin D supplementation and COVID-19 infection and mortality. Sci. Rep..

[60] Kaufman H.W., Niles J.K., Kroll M.H., Bi C., Holick M.F. (2020). SARS-CoV-2 positivity rates associated with circulating 25-hydroxyvitamin D levels. PLoS ONE.

[61] Sîrbe C., Rednic S., Grama A., Pop T.L. (2022). An Update on the Effects of Vitamin D on the Immune System and Autoimmune Diseases. Int. J. Mol. Sci..

[62] McDonnell S.L., Baggerly C., French C.B., Baggerly L.L., Garland C.F., Gorham E.D., Lappe J.M., Heaney R.P. (2016). Serum 25-Hydroxyvitamin D Concentrations ≥40 ng/ml Are Associated with >65% Lower Cancer Risk: Pooled Analysis of Randomized Trial and Prospective Cohort Study. PLoS ONE.

[63] Ames B.N., Grant W.B., Willett W.C. (2021). Does the High Prevalence of Vitamin D Deficiency in African Americans Contribute to Health Disparities?. Nutrients.

[64] Morales-Suárez-Varela M., Uçar N., Soriano J.M., Llopis-Morales A., Sanford B.S., Grant W.B. (2022). Vitamin D-Related Risk Factors for Maternal Morbidity and Mortality during Pregnancy: Systematic Review and Meta-Analysis. Nutrients.

[65] Suárez-Varela M.M., Uçar N., Peraita-Costa I., Huertas M.F., Soriano J.M., Llopis-Morales A., Grant W.B. (2022). Vitamin D-Related Risk Factors for Maternal Morbidity during Pregnancy: A Systematic Review. Nutrients.

[66] Rostami M., Tehrani F.R., Simbar M., Bidhendi Yarandi R., Minooee S., Hollis B.W., Hosseinpanah F. (2018). Effectiveness of Prenatal Vitamin D Deficiency Screening and Treatment Program: A Stratified Randomized Field Trial. J. Clin. Endocrinol. Metab..

[67] Kemény L.V., Robinson K.C., Hermann A.L., Walker D.M., Regan S., Yew Y.W., Lai Y.C., Theodosakis N., Rivera P.D., Ding W. (2021). Vitamin D deficiency exacerbates UV/endorphin and opioid addiction. Sci. Adv..

[68] Akkus M., Davarci P.Z., Bas S., Odluyurt H., Aydogan M. (2022). Evaluation of inflammatory parameters in patients who attempted suicide by taking drugs. Bratisl. Lek. Listy..

[69] Lavigne J.E., Gibbons J.B. (2023). The association between vitamin D serum levels, supplementation, and suicide attempts and intentional self-harm. PLoS ONE.

[70] Kouba B.R., Camargo A., Gil-Mohapel J., Rodrigues A.L.S. (2022). Molecular Basis Underlying the Therapeutic Potential of Vitamin D for the Treatment of Depression and Anxiety. Int. J. Mol. Sci..

[71] Kouba B.R., Torrá A.C.N.C., Camargo A., Rodrigues A.L.S. (2023). The antidepressant-like effect elicited by vitamin D3 is associated with BDNF/TrkB-related synaptic protein synthesis. Metab. Brain Dis..

[72] Mikola T., Marx W., Lane M.M., Hockey M., Loughman A., Rajapolvi S., Rocks T., O’neil A., Mischoulon D., Valkonen-Korhonen M. (2022). The effect of vitamin D supplementation on depressive symptoms in adults: A systematic review and meta-analysis of randomized controlled trials. Crit. Rev. Food Sci. Nutr..

[73] Somoza-Moncada M.M., Turrubiates-Hernández F.J., Muñoz-Valle J.F., Gutiérrez-Brito J.A., Díaz-Pérez S.A., Aguayo-Arelis A., Hernández-Bello J. (2023). Vitamin D in Depression: A Potential Bioactive Agent to Reduce Suicide and Suicide Attempt Risk. Nutrients.

[74] Umhau J.C., George D.T., Heaney R.P., Lewis M.D., Ursano R.J., Heilig M., Hibbeln J.R., Schwandt M.L. (2013). Low Vitamin D Status and Suicide: A Case-Control Study of Active Duty Military Service Members. PLoS ONE.

[75] Yagci I., Avci S. (2021). Biochemical predictors in presentations to the emergency department after a suicide attemp. Bratisl. Lek. Listy..

[76] Zubizarreta J.R., Umhau J.C., Deuster P.A., Brenner L.A., King A.J., Petukhova M.V., Sampson N.A., Tizenberg B., Upadhyaya S.K., RachBeisel J.A. (2022). Evaluating the heterogeneous effect of a modifiable risk factor on suicide: The case of vitamin D deficiency. Int. J. Methods Psychiatr. Res..

[77] Melough M.M., Li M., Hamra G., Palmore M., Sauder K.A., Dunlop A.L., LeWinn K.Z., Zhao Q., Kelly R.S., Switkowski K.M. (2023). Greater Gestational Vitamin D Status is Associated with Reduced Childhood Behavioral Problems in the Environmental Influences on Child Health Outcomes Program. J. Nutr..

[78] Moukayed M. (2023). A Narrative Review on the Potential Role of Vitamin D3 in the Prevention, Protection, and Disease Mitigation of Acute and Long COVID-19. Curr. Nutr. Rep..

[79] Gonzalez S.M., Aguilar-Jimenez W., Trujillo-Gil E., Zapata W., Su R.-C., Ball T.B., Rugeles M.T. (2019). Vitamin D treatment of peripheral blood mononuclear cells modulated immune activation and reduced susceptibility to HIV-1 infection of CD4+ T lymphocytes. PLoS ONE.

[80] Pittas A.G., Dawson-Hughes B., Sheehan P., Ware J.H., Knowler W.C., Aroda V.R., Brodsky I., Ceglia L., Chadha C., Chatterjee R. (2019). Vitamin D Supplementation and Prevention of Type 2 Diabetes. N. Engl. J. Med..

[81] McDonnell S.L., Baggerly C.A., French C.B., Baggerly L.L., Garland C.F., Gorham E.D., Hollis B.W., Trump D.L., Lappe J.M. (2018). Breast cancer risk markedly lower with serum 25-hydroxyvitamin D concentrations ≥60 vs <20 ng/ml (150 vs. 50 nmol/L): Pooled analysis of two randomized trials and a prospective cohort. PLoS ONE.

[82] Saeed A.A., Eid M., Ahmed S., Abboud M., Sami B. (2020). Knowledge, attitude, and practice regarding Vitamin D deficiency among community pharmacists and prescribing doctors in Khartoum city, Sudan, 2020. Matrix Sci. Pharma..

[83] Farooq M., Mohamedally S., Doshani A., Mousa H. (2014). PMM.61 Are doctors well informed on Vitamin D and its’ prescribing during pregnancy?. Arch. Dis. Child. Fetal Neonatal Ed..

[84] Simon A.E., Ahrens K.A. (2020). Adherence to Vitamin D Intake Guidelines in the United States. Pediatrics.

[85] Ross E.J., Fitzpatrick J.J., Click E.R., Krouse H.J., Clavelle J.T. (2014). Transformational leadership practices of nurse leaders in professional nursing associations. J. Nurs. Adm..

[86] Titler M.G., Hughes R.G. (2008). The Evidence for Evidence-Based Practice Implementation. Patient Safety and Quality: An. Evidence-Based Handbook for Nurses.

[87] Bandura A. (1986). Social Foundations of Thought and Action: A Social Cognitive Theory.

[88] GrassrootsHealth (2023). Moving Vitamin D Research into Practice: Addressing Vitamin D Deficiency to Improve Patient Outcomes, Population Health & Reduce Costs.

[89] Golden T.L., Wendel M.L. (2020). Public Health’s Next Step in Advancing Equity: Re-evaluating Epistemological Assumptions to Move Social Determinants From Theory to Practice. Front. Public Health.

[90] MUSC Health (2017). Vitamin D Testing and Treatment Protocol. https://www.grassrootshealth.net/wp-content/uploads/2017/10/newman-protocol-letter.pdf?_ga=2.20709048.1817993166.1668455725-1671503586.1657558075.

[91] Screening, Brief Intervention, and Referral to Treatment (SBIRT). https://www.samhsa.gov/sbirt.

[92] Sanford B., Aliano J. Cycle of Best Practices for Addressing Vitamin D Deficiency. 2022. https://www.researchgate.net/publication/366189944_Cycle_of_Best_Practices_for_Addressing_Vitamin_D_Deficiency?channel=doi&linkId=6396913d095a6a7774229362&showFulltext=true.

[93] Wagner C.L., Baggerly C., McDonnell S., Baggerly K.A., French C.B., Baggerly L., Hamilton S.A., Hollis B.W. (2016). Post-hoc analysis of vitamin D status and reduced risk of preterm birth in two vitamin D pregnancy cohorts compared with South Carolina March of Dimes 2009-2011 rates. J. Steroid Biochem. Mol. Biol..

[94] (2021). Why the Same Dose Does Not Work for Everyone.

[95] Kimball S.M., Holick M.F. (2020). Official recommendations for vitamin D through the life stages in developed countries. Eur. J. Clin. Nutr..

[96] Crowe F.L., Steur M., Allen N.E., Appleby P.N., Travis R.C., Key T.J. (2011). Plasma concentrations of 25-hydroxyvitamin D in meat eaters, fish eaters, vegetarians and vegans: Results from the EPIC–Oxford study. Public. Health Nutr..

[97] (2022). Everyone Responds Differently to Vitamin D Infographic.

[98] (2022). Vitamin D Deficiency Risk Assessment Quiz.

[99] GrassrootsHealth (2020). Vitamin D*calculatorTM.

[100] (2022). KNOW “D” NUMBER Patient and Provider Guide to Understanding Vitamin D, Testing & Results Booklet.

[101] Create Courses Online|#1 E-learning Software Platform Create an online course easily|Easygenerator. https://www.easygenerator.com/en/.

[102] Khammarnia M., Haj Mohammadi M., Amani Z., Rezaeian S., Setoodehzadeh F. (2015). Barriers to implementation of evidence based practice in zahedan teaching hospitals, iran, 2014. Nurs. Res. Pract..

[103] (2022). Achieve and Manage Your Optimal Vitamin D Levels.

[104] (2020). NEW Loading Dose Vitamin D*Calculator!.

[105] van Groningen L., Opdenoordt S., van Sorge A., Telting D., Giesen A., de Boer H. (2010). Cholecalciferol loading dose guideline for vitamin D-deficient adults. Eur. J. Endocrinol..

[106] Engelsen O. (2010). The Relationship between Ultraviolet Radiation Exposure and Vitamin D Status. Nutrients.

[107] Engelsen O., Brustad M., Aksnes L., Lund E. (2005). Daily Duration of Vitamin D Synthesis in Human Skin with Relation to Latitude, Total Ozone, Altitude, Ground Cover, Aerosols and Cloud Thickness. Photochem. Photobiol..

[108] Estupiñán J.G., Raman S., Crescenti G.H., Streicher J.J., Barnard W.F. (1996). Effects of Clouds and Haze on UV-B Radiation. J. Geophys. Res..

[109] Leal A.C.G.B., Corrêa M.P., Holick M.F., Melo E.V., Lazaretti-Castro M. (2021). Sun-induced production of vitamin D3 throughout 1 year in tropical and subtropical regions: Relationship with latitude, cloudiness, UV-B exposure and solar zenith angle. Photochem. Photobiol. Sci..

[110] Heaney R.P. (2014). Guidelines for optimizing design and analysis of clinical studies of nutrient effects. Nutr. Rev..

