# Supplementary material for: Exposure to a Vitamin D Best Practices Toolkit, Model, and E-Tools Increases Knowledge, Confidence, and the Translation of Research to Public Health and Practice
Source: Nutrients. 2023 May 24;15(11):2446. doi: 10.3390/nu15112446 (PMC10254851; doi:10.3390/nu15112446)
Supplement: Supplementary file 1 [file nutrients-15-02446-s001.zip › nutrients-2377239-supplementary.pdf]

Table S1: Vitamin D Resources for Healthcare Professionals

**Peer-Reviewed Journal Articles**	
Call to Public Health Action	Baggerly, C. A., Cuomo, R. E., French, C. B., Garland, C. F., Gorham, E. D., Grant, W. B., Heaney, R. P., Holick, M. F., Hollis, B. W., McDonnell, S. L., Pittaway, M., Seaton, P., Wagner, C. L., & Wunsch, A. (2015). Sunlight and vitamin D: Necessary for public health. *Journal of the American College of Nutrition*, *34*(4), 359–365. https://doi.org/10.1080/07315724.2015.1039866<br><br>Grant, W.B., Al Anouti, F. & Moukayed, M. (2020). Targeted 25-hydroxyvitamin D concentration measurements and vitamin D_3 supplementation can have important patient and public health benefits. *Eur J Clin Nutr,* **74,** 366–376 https://doi.org/10.1038<br><br>Holick M. F. (2017). The vitamin D deficiency pandemic: Approaches for diagnosis, treatment, and prevention. *Reviews in Endocrine & Metabolic Disorders*, *18*(2), 153–165. https://doi.org/10.1007/s11154-017-9424-1<br><br>Kimball, S. M., & Holick, M. F. (2020). Official recommendations for vitamin D through the Life stages in developed countries. *European Journal of Clinical Nutrition*, *74*(11), 1514–1518. https://doi.org/10.1038/s41430-020-00706-3
Research Appraisal	Heaney R. P. (2014). Guidelines for optimizing design and analysis of clinical studies of nutrient effects. *Nutrition reviews*, *72*(1), 48–54. https://doi.org/10.1111/nure.12090<br><br>Hollis, B. W., & Wagner, C. L. (2013). Clinical review: The role of the parent compound vitamin D with respect to metabolism and function: Why clinical dose intervals can affect clinical outcomes. *The Journal of Clinical Endocrinology and Metabolism*, *98*(12), 4619–4628. https://doi.org/10.1210/jc.2013-2653<br><br>Pilz, S., Trummer, C., Theiler-Schwetz, V., Grübler, M. R., Verheyen, N. D., Odler, B., Karras, S. N., Zittermann, A., & März, W. (2022). Critical Appraisal of Large Vitamin D Randomized Controlled Trials. *Nutrients*, *14*(2), 303. https://doi.org/10.3390/nu14020303<br><br>Infographic to aid with research appraisal https://www.grassrootshealth.net/document/assess-validity-vitamin-d-research-infographic/
Vitamin D Physiology	Heaney, R. P., & Armas, L. A. (2015). Quantifying the vitamin D economy. *Nutrition reviews*, *73*(1), 51–67. https://doi.org/10.1093/nutrit/nuu004<br><br>Holick M. F. (2007). Vitamin D deficiency. *The New England Journal of Medicine*, *357*(3), 266–281. https://doi.org/10.1056/NEJMra070553
Totality of Evidence	Grant, W. B., Boucher, B. J., Al Anouti, F., & Pilz, S. (2022). Comparing the Evidence from Observational Studies and Randomized Controlled Trials for Nonskeletal Health Effects of Vitamin D. *Nutrients*, *14*(18), 3811. https://doi.org/10.3390/nu14183811<br><br>Grant, W. B., Al Anouti, F., Boucher, B. J., Fakhoury, H. M. A., Moukayed, M., Pilz, S., & Al-Daghri, N. M. (2023). Evidence That Increasing Serum 25(OH)D Concentrations to 30 ng/mL in the Kingdom of Saudi Arabia and the United Arab Emirates Could Greatly Improve Health Outcomes. *Biomedicines*, *11*(4), 994. MDPI AG. Retrieved from http://dx.doi.org/10.3390/biomedicines11040994
General Vitamin D meta-analyses	Grant, W. B., Al Anouti, F., Boucher, B. J., Dursun, E., Gezen-Ak, D., Jude, E. B., Karonova, T., & Pludowski, P. (2022). A narrative review of the evidence for variations in serum 25-

	Hydroxyvitamin D concentration thresholds for optimal health. *Nutrients*, *14*(3), 639. https://doi.org/10.3390/nu14030639
Cardiovascular Disease	Acharya, P., Dalia, T., Ranka, S., Sethi, P., Oni, O. A., Safarova, M. S., Parashara, D., Gupta, K., & Barua, R. S. (2021). The Effects of Vitamin D Supplementation and 25-Hydroxyvitamin D Levels on the Risk of Myocardial Infarction and Mortality. *Journal of the Endocrine Society*, *5*(10), bvab124. https://doi.org/10.1210/jendso/bvab124
Type 2 Diabetes	McDonnell, S. L., Baggerly, L. L., French, C. B., Heaney, R. P., Gorham, E. D., Holick, M. F., Scragg, R., & Garland, C. F. (2016). Incidence rate of type 2 diabetes is >50% lower in GrassrootsHealth cohort with median serum 25-hydroxyvitamin D of 41 ng/ml than in NHANES cohort with median of 22 ng/ml. *The Journal of Steroid Biochemistry and Molecular Biology*, *155*(Pt B), 239–244. https://doi.org/10.1016/j.jsbmb.2015.06.013<br><br>Chatterjee, R., Davenport, C. A., Vickery, E. M., Johnson, K. C., Kashyap, S. R., LeBlanc, E. S., Nelson, J., Dagogo-Jack, S., Pittas, A. G., Hughes, B. D., D2d Research Group, Steering Committee, Past Steering Committee members, & Advisor (2023). Effect of intra-trial mean 25(OH)D level on diabetes risk, by race and weight: an ancillary analysis in the D2d randomized study cohort. *The American journal of clinical nutrition*, S0002-9165(23)46304-6. Advance online publication. https://doi.org/10.1016/j.ajcnut.2023.03.021
Cancer	Muñoz, A., & Grant, W. B. (2022). Vitamin D and cancer: An historical overview of the epidemiology and mechanisms. *Nutrients*, *14*(7), 1448. https://doi.org/10.3390/nu14071448
Autoimmune Disease	Mirhosseini, N., Brunel, L., Muscogiuri, G., & Kimball, S. (2017). Physiological serum 25-hydroxyvitamin D concentrations are associated with improved thyroid function-observations from a community-based program. *Endocrine*, *58*(3), 563–573. https://doi.org/10.1007/s12020-017-1450-y
COVID-19	Gibbons, J. B., Norton, E. C., McCullough, J. S., Meltzer, D. O., Lavigne, J., Fiedler, V. C., & Gibbons, R. D. (2022). Association between vitamin D supplementation and COVID-19 infection and mortality. *Scientific reports*, *12*(1), 19397. https://doi.org/10.1038/s41598-022-24053-4<br><br>Kaufman, H. W., Niles, J. K., Kroll, M. H., Bi, C., & Holick, M. F. (2020). SARS-CoV-2 positivity rates associated with circulating 25-hydroxyvitamin D levels. *PloS one*, *15*(9), e0239252. https://doi.org/10.1371/journal.pone.0239252<br><br>Moukayed M. (2023). A Narrative Review on the Potential Role of Vitamin D_3 in the Prevention, Protection, and Disease Mitigation of Acute and Long COVID-19. *Current nutrition reports*, 10.1007/s13668-023-00471-2. Advance online publication. https://doi.org/10.1007/s13668-023-00471-2
Pregnancy/Infant Outcomes	Hollis, B. W., Wagner, C. L., Howard, C. R., Ebeling, M., Shary, J. R., Smith, P. G., Taylor, S. N., Morella, K., Lawrence, R. A., & Hulsey, T. C. (2015). Maternal versus infant vitamin D supplementation during lactation: A randomized controlled trial. *Pediatrics*, *136*(4), 625–634. https://doi.org/10.1542/peds.2015-1669<br><br>McDonnell, S. L., Baggerly, K. A., Baggerly, C. A., Aliano, J. L., French, C. B., Baggerly, L. L., Ebeling, M. D., Rittenberg, C. S., Goodier, C. G., Mateus Niño, J. F., Wineland, R. J., Newman, R. B., Hollis, B. W., & Wagner, C. L. (2017). Maternal 25(OH)D concentrations ≥40 ng/mL associated with 60% lower preterm birth risk among general obstetrical patients at an urban medical center. *PloS One*, *12*(7), e0180483. https://doi.org/10.1371/journal.pone.0180483<br><br>Rostami, M., Tehrani, F. R., Simbar, M., Bidhendi Yarandi, R., Minooee, S., Hollis, B. W., & Hosseinpanah, F. (2018). Effectiveness of Prenatal Vitamin D Deficiency Screening and Treatment Program: A Stratified Randomized Field Trial. *The Journal of clinical endocrinology and metabolism*, *103*(8), 2936–2948. https://doi.org/10.1210/jc.2018-00109

Mental/Cognitive Health	Kouba, B. R., Camargo, A., Gil-Mohapel, J., & Rodrigues, A. (2022). Molecular Basis Underlying the Therapeutic Potential of Vitamin D for the Treatment of Depression and Anxiety. *International journal of molecular sciences*, *23*(13), 7077. https://doi.org/10.3390/ijms23137077<br><br>Lavigne, J. E., & Gibbons, J. B. (2023). The association between vitamin D serum levels, supplementation, and suicide attempts and intentional self-harm. *PloS one*, *18*(2), e0279166. https://doi.org/10.1371/journal.pone.0279166<br><br>Mikola, T., Marx, W., Lane, M. M., Hockey, M., Loughman, A., Rajapolvi, S., Rocks, T., O'Neil, A., Mischoulon, D., Valkonen-Korhonen, M., Lehto, S. M., & Ruusunen, A. (2022). The effect of vitamin D supplementation on depressive symptoms in adults: A systematic review and meta-analysis of randomized controlled trials. *Critical reviews in food science and nutrition*, 1–18. https://doi.org/10.1080/10408398.2022.2096560
Musculoskeletal Health	Grant, W. B., Al Anouti, F., Boucher, B. J., Fakhoury, H. M. A., Moukayed, M., Pilz, S., & Al-Daghri, N. M. (2023). Evidence That Increasing Serum 25(OH)D Concentrations to 30 ng/mL in the Kingdom of Saudi Arabia and the United Arab Emirates Could Greatly Improve Health Outcomes. *Biomedicines*, *11*(4), 994. MDPI AG. Retrieved from http://dx.doi.org/10.3390/biomedicines11040994
Gastrointestinal Health	Fakhoury, H., Kvietys, P. R., AlKattan, W., Anouti, F. A., Elahi, M. A., Karras, S. N., & Grant, W. B. (2020). Vitamin D and intestinal homeostasis: Barrier, microbiota, and immune modulation. *The Journal of Steroid Biochemistry and Molecular Biology*, *200*, 105663. https://doi.org/10.1016/j.jsbmb.2020.105663
Dental Health	Uwitonze, A. M., Murererehe, J., Ineza, M. C., Harelimana, E. I., Nsabimana, U., Uwambaye, P., Gatarayiha, A., Haq, A., & Razzaque, M. S. (2018). Effects of vitamin D status on oral health. *The Journal of steroid biochemistry and molecular biology*, *175*, 190–194. https://doi.org/10.1016/j.jsbmb.2017.01.020<br><br>Grant, W. B., & Boucher, B. J. (2010). Are Hill's criteria for causality satisfied for vitamin D and periodontal disease? *Dermato-endocrinology*, *2*(1), 30–36. https://doi.org/10.4161/derm.2.1.12488
Cost-Effectiveness	Lacey, L. F., Armstrong, D. J., Royle, E., Magee, P., Pourshahidi, L. K., Ray, S., Strain, J. J., & McSorley, E. (2022). Cost-effectiveness of vitamin D_3 supplementation in older adults with vitamin D deficiency in Ireland. *BMJ nutrition, prevention & health*, *5*(1), 98–105. https://doi.org/10.1136/bmjnph-2021-000382<br><br>Peiris, A. N., Bailey, B. A., & Manning, T. (2008). The relationship of vitamin D deficiency to health care costs in veterans. *Military Medicine*, *173*(12), 1214–1218. https://doi.org/10.7205/milmed.173.12.1214

**Resource Type**	**Title**	**Location**
CEU--Vitamin D Healthcare Professional Education e-Course	*Moving Vitamin D Research into Practice: Addressing Vitamin D Deficiency to Improve Patient Outcomes, Population Health & Reduce Costs*	Announcing a New Vitamin D*Education Course - Great for Everyone, and CEU Approved! - GrassrootsHealth
Translational Model	*The Cycle of Best Practices for Addressing Vitamin D Deficiency*	Sanford, B. S. & Aliano, J. L. (2022). Cycle of best practices for addressing vitamin D deficiency. (19) (PDF) Cycle of Best Practices for Addressing Vitamin D Deficiency (researchgate.net)

Translational Resource Guide	The IRB-approved *KNOW "D" NUMBER Patient and Provider Guide*	https://www.grassrootshealth.net/document/know-d-number-patient-provider-guide-understanding-vitamin-d-testing-results-booklet/KNOW "D" NUMBER Patient and Provider Guide to Understanding Vitamin D, Testing & Results Booklet - GrassrootsHealth
IRB-approved e-tool (beta)	*Vitamin D Deficiency Risk Assessment Quiz*	GrassrootsHealth. (2022). *Achieve and manage your optimal vitamin D levels*. GrassrootsHealth Nutrient Research Institute. https://grassrootshealth.org/project/achieve-manage-optimal-vitamin-d-levels/
IRB-approved e-tool (2015)	*Vitamin D*Calculator™*	GrassrootsHealth. (2022). *Achieve and manage your optimal vitamin D levels*. GrassrootsHealth Nutrient Research Institute. https://grassrootshealth.org/project/achieve-manage-optimal-vitamin-d-levels/
Infographics	Vitamin D and Nutrients Infographics for HCP or patient education, and social media	https://www.grassrootshealth.net/blog/new-infographics-documentation-page/NEW! Infographics Documentation Page - GrassrootsHealth
Vitamin D and Nutrient Science Newsletter	GrassrootsHealth Newsletter	GrassrootsHealth : Sign Up to Stay Informed on Vitamin D and Nutrient Research
Organizations	GrassrootsHealth Nutrient Research Institute<br><br>Vitamin D Society of Canada<br><br>SUNARC	Home - GrassrootsHealth<br><br>The VitaminD Society<br><br>SUNARC - Sunlight, Nutrition, and Health Research Center | Vitamin D for Better Health
Websites	Vitamin D Wiki – contains a library of vitamin D research	https://vitamindwiki.com/
